# Computational Mechanisms of Learning and Forgetting Differentiate Affective and Substance Use Disorders

**DOI:** 10.21203/rs.3.rs-4682224/v1

**Published:** 2024-10-31

**Authors:** Navid Hakimi, Ko-Ping Chou, Jennifer L. Stewart, Martin P. Paulus, Ryan Smith

**Affiliations:** 1 Laureate Institute for Brain Research, Tulsa, OK.; 2 Oxley College of Health and Natural Sciences, University of Tulsa, Tulsa, OK.

**Keywords:** affective disorders, substance use disorders, computational psychiatry, active inference, decision-making, explore-exploit dilemma, reinforcement learning

## Abstract

Depression and anxiety are common, highly co-morbid conditions associated with a range of learning and decision-making deficits. While the computational mechanisms underlying these deficits have received growing attention, the transdiagnostic vs. diagnosis-specific nature of these mechanisms remains insufficiently characterized. Individuals with affective disorders (iADs; i.e., depression with or without co-morbid anxiety; N=168 and 74, respectively) completed a widely-used decision-making task. To establish diagnostic specificity, we also incorporated data from a sample of individuals with substance use disorders (iSUDs; N=147) and healthy comparisons (HCs; N=54). Computational modeling afforded separate measures of learning and forgetting rates, among other parameters. Compared to HCs, forgetting rates (reflecting recency bias) were elevated in both iADs and iSUDs (*p* = 0.007, *η*^*2*^ = 0.022). In contrast, iADs showed faster learning rates for negative outcomes than iSUDs (*p* = 0.027, *η*^*2*^ = 0.017), but they did not differ from HCs. Other model parameters associated with learning and information-seeking also showed suggestive relationships with early adversity and impulsivity. Our findings demonstrate distinct differences in learning and forgetting rates between iSUDs, iADs, and HCs, suggesting that different cognitive processes are affected in these conditions. These differences in decision-making processes and their correlations with symptom dimensions suggest that one could specifically develop interventions that target changing forgetting rates and/or learning from negative outcomes. These results pave the way for replication studies to confirm these relationships and establish their clinical implications.

## Introduction

Depression and anxiety are among the most prevalent and debilitating disorders worldwide ^1^. They are also highly co-morbid ^2^ and known to have a profound negative impact on physical health, social and occupational functioning, and overall quality of life ^3,4^. Unfortunately, current treatments remain only partially efficacious, motivating the need to better understand underlying mechanisms. Some potentially relevant cognitive mechanisms in this context involve learning and decision-making under uncertainty, which may be affected in different ways within affective disorders ^5–7^. The heterogeneous nature of these disorders has also highlighted the potential importance of identifying and targeting such mechanisms on an individual basis. At present, however, learning and decision-making processes in affective disorders remain only partially characterized, and diagnostic specificity has not been established, which may be especially important for informing treatment selection.

Recently, the emerging field of computational psychiatry has played a major role in improving our understanding of these processes ^8,9^. By fitting models of the information processing mechanisms underlying choice behavior, computational methods offer the promise of identifying individual differences in such mechanisms and their relationship to maladaptive decision-making patterns. This can then serve a range of purposes, including development of objective severity markers, accounting for heterogeneity within and between diagnoses, and predicting treatment response, among others ^10,11^. However, considerable progress is still needed before such potential applications can be realized. Given the growing appreciation for heterogeneity in the underlying causes of affective disorders, one key factor that will require increased attention is whether particular computational mechanisms reflect common transdiagnostic factors or whether some may be diagnosis-specific and allow differentiation from other conditions. For example, altered learning and decision-making mechanisms have also been consistently observed in individuals with substance use disorders ^12,13^, with or without co-morbid affective disorders. It remains undetermined, however, whether specific mechanisms differentiate these two types of disorders.

Here there is some reason to suspect at least partially unique mechanisms. For example, anxiety, depression, and substance use disorders have each been associated with altered learning rates in reinforcement learning (RL) models, but the nature of these effects may differ between groups. Anxiety and stress have often been linked to elevated and inflexible learning rates generally ^14–18^, while depression may be associated with faster learning from negative outcomes in particular ^19^. Substance use disorders have instead been linked to slower learning from negative outcomes and reduced punishment sensitivity ^20–25^. Another potentially important decision-making mechanism pertains to the drive to reduce uncertainty. In this case, there is mixed evidence across clinical groups suggesting either avoidance of uncertain options that would afford information gain (i.e., under-exploration) or exaggerated and persistent attempts to reduce uncertainty (i.e., over-exploration), where results appear to depend on choice of task, symptom measures, and/or specific disorder subtype ^26–31^. While these examples suggest the power to potentially differentiate clinical groups, direct comparison between affective disorders and other conditions has only been done in a few studies ^32–34^, and differential diagnostic information has not been the focus of this prior work.

In the present study, we sought to address these issues in a sample of individuals with affective disorders (iADs; depression with or without co-morbid anxiety disorders) who completed an established decision-making task often used with computational modeling. We compared this group of individuals to previously published data on individuals with substance use disorders (iSUDs) and healthy comparisons (HCs)^20^. This allowed us to test whether computational mechanisms associated with learning and decision-making under uncertainty were affected similarly across disorder groups relative to healthy individuals, or whether some mechanisms might differentiate iADs and iSUDs. It also allowed comparison of depression with vs. without anxiety disorders and assessment of associations with symptom severity. Our overarching goal was to offer direct comparison of an affective disorders group to a clinical control group also known to show decision-making deficits and thereby establish the clinical generality or specificity of these computational mechanisms. This was considered an important first step in highlighting factors that may be of specific relevance to understanding and treating affective disorders. As described below, our modeling approach also allowed us to distinguish distinct aspects of belief updating associated with recency bias (e.g., jumping to conclusions based on a single new observation) and differential sensitivity to negative and positive outcomes, each of which map on to common features of psychopathology. Based on the work reviewed above, we hypothesized that, compared to both HCs and iSUDs, those with iADs would show a greater pessimism (reducing exploratory choices), greater learning rates from negative outcomes (increasing punishment sensitivity) and greater forgetting rates (i.e., recency bias, reducing confidence in previous experience), reflecting less trust in the predictability of the environment.

## Methods

### Participants

Individuals in this study were sampled from the exploratory dataset (initial 500 participants) of the Tulsa 1000 (T1000) project ^35^. The T1000 is a naturalistic, longitudinal cohort study, which recruited participants based on the NIMH Research Domain Criteria (RDoC) framework. Participants were age 18–55 and screened based on dimensional psychopathology measures: Patient Health Questionnaire for depression (PHQ-9; ^36^) scores ≥ 10, Overall Anxiety Severity and Impairment Scale (OASIS; ^37^) scores ≥ 8, and Drug Abuse Screening Test (DAST-10 (Bohn et al., 1991)) scores ≥ 3. The iADs group included 74 individuals diagnosed with major depressive disorder alone (iDEP) and 168 with co-morbid depression and anxiety disorders (iDEP/ANX), where anxiety disorders could include generalized anxiety disorder, panic disorder, social anxiety disorder, and/or post-traumatic stress disorder. The iSUDs group included those with alcohol, nicotine, cannabis, sedative, stimulant, hallucinogen, and/or opioid use disorders (however, alcohol or nicotine use disorders alone were not sufficient for inclusion in the larger T1000 study), who were also allowed to meet criteria for major depressive disorder and anxiety disorders. HCs in this sample did not meet criteria for any diagnosis and fell below thresholds for all screening measures above. Exclusion criteria included: (a) a positive urine drug test, (b) diagnosis of psychotic, bipolar, or obsessive-compulsive disorders, (c) prior record of moderate-to-severe traumatic brain injuries, neurological issues, or other significant medical conditions, (d) active suicidality, or (e) medication adjustments in the past 6 weeks. Detailed criteria for inclusion and exclusion can be found in Victor, et al. ^35^. The study protocol received approval from the WCG Institutional Review Board and adhered to all ethical guidelines established by the 1964 declaration of Helsinki. All participants gave their written consent before participating in the study and received compensation; ClinicalTrials.gov identifier: #NCT02450240. Table 1 summarizes the demographics and clinical characteristics of all participants included in the present study. Supplementary Table S1 summarizes the breakdown of comorbidities in the affective disorders group. Supplementary Table S2 shows the breakdown of comorbidities within the substance used disorders group.

### Decision-Making Task

Primary behavioral measures were derived from a commonly used Three-Armed Bandit (TAB) task ^38^. Note that data from HCs and iSUDs on this task have been reported previously ^20^. Here we use these participants as normative and clinical comparison groups (respectively) when evaluating previously unanalyzed data in iADs. However, as described below, application of novel computational models here also afforded novel measures across participants in all groups.

In brief, this task has 20 blocks and 16 trials per block. One each block, participants are asked to repeatedly choose between three options, each associated with a fixed probability of reward. These probabilities are stable within each block but change between blocks. Thus, they must be learned on each block through trial and error. The task is designed to examine the strategic interplay between exploration (trying to identify the best option) and exploitation (repeatedly choosing the option currently believed to be best).

### Behavioral measures

Participants’ task performance was assessed using several model-free metrics. These included the total number of wins, mean reaction times, and behavioral strategies indicated by the proportion of trials participants chose the same option after a win (win-stay) and the proportion of trials they chose a different option after a loss (lose-shift). Outliers were removed based on an iterative Grubbs’ test ^39^ with a threshold of *p*=0.01. Linear models were used to test for potential differences between groups (HCs, iADs, iSUDs) for each of these metrics, while accounting for age and sex. Post-hoc contrasts of estimated marginal means (EMMs) were then used to interpret any significant differences between groups.

### Computational Modeling

**Figure 1.**
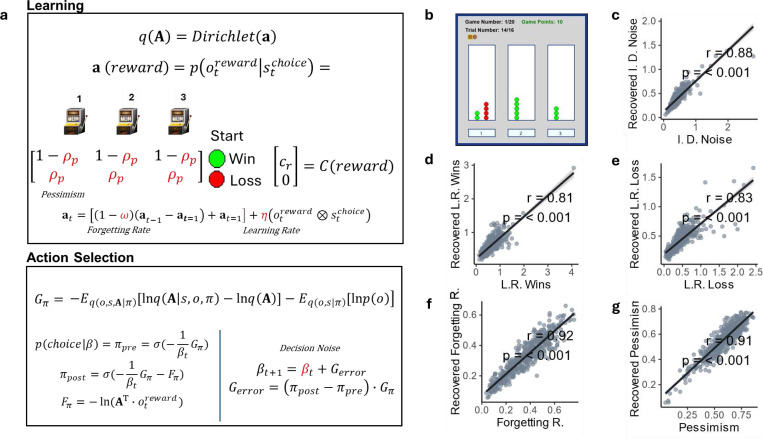
Task structure and computational model. **a)** The winning computational (active inference) model included 5 parameters: Initial Decision Noise (*β*_0_), Learning Rates for Wins (*η_win_*) and Losses (*η_loss_*), Forgetting Rate (*ω*), and Pessimism (*ρ_p_*). Higher *β*_0_ values decrease the initial precision of the probability distribution from which actions are sampled (*π*). This value then changes across trials, leading to noisier choice after unexpected negative outcomes and less noisy choice after positive outcomes. Higher learning rates increase the degree to which confidence increases about a particular action-outcome probability after each new win or loss. Higher *ω* values decrease confidence in current beliefs about all action-outcome probabilities after each trial, leading to a recency bias in learning. Higher *ρ_p_* values deter exploratory choices, as they entail that unchosen actions will likely lead to negative outcomes. Note that, in the equations shown, **A** is a matrix of action-outcome probabilities, which corresponds to the normalized columns of **a**-matrix. This latter matrix counts the number of times each outcome has been observed after each action (where these counts are scaled by learning and forgetting rates). *G_π_* assigns lower values to actions expected to both generate more reward and lead to greater reductions in uncertainty about action-outcome probabilities. *F_π_* quantifies the unexpectedness of an outcome under a chosen action, while *G_error_* quantifies how strongly beliefs about the best action change after a new observation. Note that *σ* indicates a softmax function that converts a vector of values into a proportional probability distribution. ⨂ represents matrix multiplication. **b)** Three-armed bandit task interface. Participants played 20 games of 16 trials each. **c-e)** Correlations between generative and estimated parameter values in simulations, demonstrating recoverability of each parameter in the winning model.

We compared a total of 15 models, including 7 Reinforcement Learning (RL) models and 8 Bayesian (Active Inference; AI) models. Full descriptions of each model are provided in Supplementary Materials. Briefly, the space of RL models was based on the Rescorla Wagner algorithm and varied by inclusion of different combinations of parameters (e.g., separate learning rates for wins and losses, dynamic [prediction error-dependent] learning rates, differences in reward sensitivity, etc.). The space of AI models was based on a commonly used partially observable Markov decision process architecture and approximate Bayesian inference algorithm ^40^, and similarly varied in the number and types of parameters that were included. Parameters in each model were estimated using a common variational Bayes approach (variational Laplace; ^41^) that maximizes the log-probability of participants’ responses while incorporating a complexity cost. For more details about chosen prior means and variances for each parameter, see Supplementary Table S3. Bayesian model comparison (based on Stephan, et al. ^42^) was then used to determine the model that best accounted for participant behavior. Figure 1a shows the main equations defining the winning model; Figure 1b shows the associated task interface with which individuals made their choices. For full modeling details, see Supplementary Materials. In parameter recoverability analyses, parameter values from the winning model were used to generate simulated data. The model was then refitted to this simulated data to recover the parameters. Recoverability was quantified by the correlation between the generative and estimated parameter values.

Bayesian model comparison revealed that an AI model best accounted for the data (protected exceedance probability=1). This model included the following parameters: initial decision noise, separate learning rate for wins and losses, forgetting rate, and pessimism. The initial decision noise parameter (*β*_0_) controls the starting level of randomness in choice, where higher values indicate less deterministic (i.e., less value-sensitive) behavior. This level of randomness was modeled as evolving over time depending on the sign and magnitude of prediction error (i.e., noise increases after unexpected negative outcomes and decreases after positive outcomes). This can therefore be interpreted as random exploration ^43^. Learning rate for wins (*η_win_*) and learning rate for losses (*η_loss_*) control the belief update magnitude about reward probabilities when observing outcomes after each action. A higher value indicates that the participant is more sensitive to the outcomes and subsequent choices are more determined by the value of each prior outcome. The forgetting Rate parameter (*ω*) reduces confidence in previously learned reward probabilities, such that choices are guided more by recent vs. remote outcomes. It can therefore be thought of as a recency bias. Lastly, the pessimism parameter (*ρ_p_*) reflects the initial belief about the probability of losing for unchosen actions. A higher pessimism reduces the drive to resolve uncertainty about reward probabilities, as the probability of winning is expected to be lower. This therefore corresponds to lower levels of directed exploration ^43^.

### Statistical Analysis

As in prior work ^20–22^, a Bayesian group-level analytic approach, Parametric Empirical Bayes (PEB; ^44^), was used to assess group differences in each model parameter. This approach allows incorporation of posterior means and variances for each parameter and therefore offers advantages over frequentist approaches that only incorporate posterior means as point estimates. Separate general linear models were used to perform comparisons between each group, with age and sex included as covariates. In this approach, PEB first performs model comparison to identify a reduced group-level model (i.e., setting some predictor coefficients to zero) that best accounts for the data, and coefficients retained in the winning model can then be evaluated in terms of their posterior probability of deviating from zero. Here, we consider posterior probabilities >.75 in the winning reduced models as indicating moderate evidence, >.95 as indicating strong evidence, and >.99 as indicating very strong evidence. As there were a few potential outliers based on visual inspection, we used an iterative Grubbs’ test ^39^ (threshold: *p*=0.01) for formal outlier identification. This identified 18 participants with at least one outlier parameter value. However, these parameter values were still plausible and generated reasonable choice behavior, so we chose not to remove them from primary analyses. However, in supplementary analyses we confirmed that the pattern of results did not differ if re-performed after removing these individuals.

Supplementary frequentist analyses were also performed analogous to the PEB models, while using posterior means as point estimates. Here, separate linear models were evaluated with each computational parameter as the outcome variable and group as a categorical predictor variable (HCs, iADs, iSUDs; sum-coded), with age and sex as covariates. Post-hoc contrasts were then performed to interpret group differences in cases where this effect was significant.

We next analyzed hypothesized relationships between trial-by-trial reaction times and model-based trial-by-trial metrics of confidence. The first metric was *choice uncertainty*, which corresponds to the entropy of posterior probabilities over actions on each trial (encoding relative confidence in one action over others). We hypothesized that higher entropy would correlate with higher reaction times, as it reflects lower confidence. The second metric was a *signed belief update magnitude* (i.e., reflecting prediction error or level of surprise, see Supplementary Information) on the prior trial. We hypothesized that more negative updates would correlate with higher reaction times, as this should lead to reductions in confidence. After running correlations between each of these metrics and reaction times across trials for each participant, we used the resulting correlation coefficient for each individual as a measure of the degree to which these variables were related, and then tested whether these relationships were significantly different from zero and whether they differed between groups (accounting for age and sex).

The T1000 study collected a large number of dimensional measures to provide a deep phenotype for each individual. To utilize this data, we first performed factor analyses for purposes of dimension reduction and used resulting factor scores as continuous variables of interest. Measures included the Anxiety Sensitivity Index (ASI) ^45^, Behavioral Activation Scale (BAS) ^46^, Big Five Personality Traits ^47^, Overall Anxiety Severity and Impairment Scale (OASIS) ^48^, Positive and Negative Affect Schedule (PANAS-X) ^49^, Patient Health Questionnaire (PHQ-9) ^50^, Patient-Reported Outcomes Measurement Information System (PROMIS) physical function, anxiety, depression, fatigue, sleep disturbance, and social roles subscales ^51^, Impulsive Behavior Scale (UPPS-P) ^52^, Childhood Trauma Questionnaire (CTQ) ^53^, Multidimensional Assessment of Interoceptive Awareness (MAIA)^54^, Toronto Alexithymia Scale (TAS) ^55^, and three measures of working memory capacity from the Wechsler Adult Intelligence Scale - IV (WAIS-IV) ^56^: forward and backward digit span and number sequencing. For a detailed description of each measure, see Supplementary Materials.

Factor analysis was implemented using the R statistical software environment, with specific functions drawn from the ‘psych’ package. Before analyses, an optimal logarithmic transformation was applied to all variables (using the *optLog* package; github/kforthman/optLog/). The data were then centered and scaled. To assess the suitability of the data for factor analysis, Bartlett’s Test of Sphericity and the Kaiser-Meyer-Olkin measure were conducted ^57^. Bartlett’s Test verifies the presence of correlations among variables, while the KMO measure evaluates the adequacy of sampling. Following these preliminary tests, the number of factors was chosen based on a threshold eigenvalue>1. The factor analysis was then performed using a principal axis factoring method, with an *oblimin* rotation applied to allow for correlations between factors.

Linear models across all participants accounting for age and sex were used to test for potential associations between factor scores and each model parameter. Each model included one parameter as the outcome variable and all factor scores as joint predictor variable. Secondary analyses in each group were also performed to check for potential relationships specific to a given diagnostic category. Both Bonferroni corrected and uncorrected significance values are reported for hypothesis generating purposes.

## Results

### Task Behavior

Model-free task performance measures (*wins*, *mean reaction times*, *win-stay/lose-shift proportions*) for each group are presented in Supplementary Table S4. There were no significant differences between groups on any measure.

For the winning model, details regarding parameters, model accuracies, and average action probabilities are provided in Supplementary Table S5. The winning model accurately predicted choices on 67.43% of trials, and the average probability assigned to participant choices was 0.60 (i.e., where chance=.33). Parameter recoverability (Figure 1c-g) was robust for all five parameters within the winning model, as all demonstrated high correlations between generative and estimated values (≥0.81).

**Figure 2.**
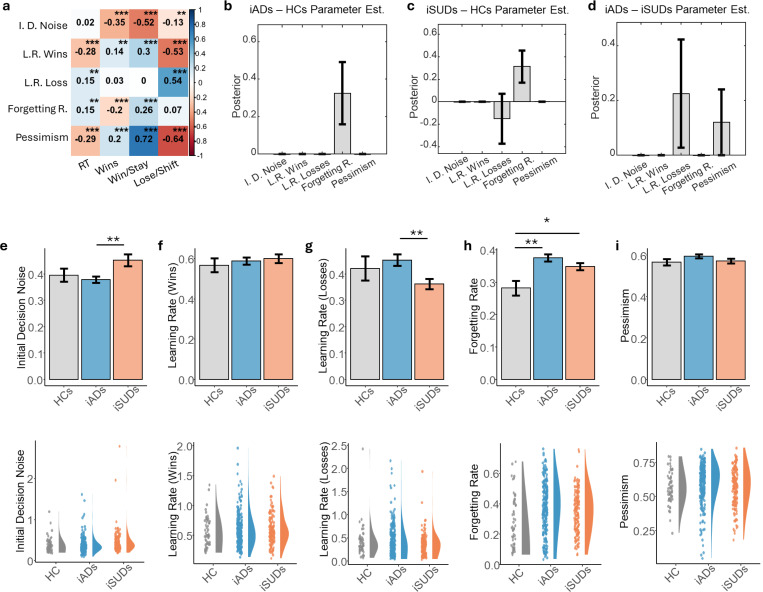
Model-free correlations and group differences in computational model parameters. **a)** Correlations between model parameters and model-free measures. **b-d)** Results of Parametric Empirical Bayes (PEB) analyses, including posterior means and credible intervals for effects of group (i.e., when accounting for effects of age and sex). Positive numbers indicate greater values in iADs relative to HCs, iSUDs relative to HCs, and iADs relative to iSUDs. Learning and forgetting rates are in logit space, while all other parameters are in log space. **e-i)** *Top*: Mean and standard error for computational parameter values in each group. Stars indicate significant differences in linear models reported in the main text (*p<.05, **p<.01, **p<.001). *Bottom*: Raincloud plot of computational model parameters for each group. RT=mean reaction time.

Correlations between model parameters and model-free measures are shown in Figure 2a. Supplementary Figure S3 also shows the inter-correlations between parameters. Relationships between model parameters and model-free measures were consistent with our previous report ^20^. Notably, learning rate for wins positively correlated with win-stay proportions and negatively correlated with lose-shift proportions. Higher initial decision noise also negatively correlated with number of wins, while higher pessimism led to fewer lose-shifts and more win-stays (i.e., consistent with less exploration). Model parameters did not show strong inter-correlations (|r|≤.23).

PEB analyses on the extended model confirmed the previously observed difference in this sample between iSUDs and HCs in learning rates for losses (i.e., lower in iSUDs; posterior probability [pp]=0.85; moderate evidence), and it also revealed differences in forgetting rates (pp=1.00; very strong evidence; Figure 2b-d), indicating slower forgetting in HCs. HCs also showed substantially slower forgetting rates than iADs in analogous models (pp=0.99), while these groups did not differ in learning rates or any other parameter. When comparing iADs to iSUDs, learning rates for losses and pessimism values were both higher in iADS (pp=0.98 and 0.96, respectively), suggesting more responsivity to negative outcomes and greater uncertainty avoidance (less directed exploration) in affective disorders, respectively.

Follow-up analyses tested for potential differences within iADs, comparing those with depression that did vs. did not have co-morbid anxiety. Here, PEB analyses suggested greater values in anxious depression in the winning group-level model for initial decision noise (pp=0.93), learning rates for wins (pp=0.87), and learning rates for losses (pp=0.92) (Supplementary Figure S1a). When instead comparing iSUDs with vs. without affective disorders, the winning group-level model retained group effects suggesting potentially lower values in those with affective disorders for initial decision noise (pp=0.79), learning rate for wins (pp=0.97), and pessimism (pp=0.94) (Supplementary Figure S2a).

Consistent with the PEB results, frequentist analyses comparing each group within a single ANOVA also indicated differences in learning rates for losses (*F*(2,438)=3.660, *p*=0.027), with iSUDs showing slower learning rates for losses than iADs (*c*=0.090, *t*=2.706, *p*=0.007). Forgetting rates also showed significant differences between groups (*F*(2,438)=5.013, *p*=0.007), with faster forgetting in both iADs (*c*=−0.072, *t*=−3.163, *p*=0.002) and iSUDs (*c*=−0.060, *t*=−2.510, p=0.012) compared to HCs. Unlike PEB results, a significant difference in initial decision noise between groups was also detected (*F*(2,438)=4.134, p=0.017) (Figure 2e-i), reflecting higher initial decision noise in iSUDs compared to iADs (*c*=−0.065, *t*=−2.808, *p*=0.005). No significant differences were observed for pessimism or learning rates for wins. Table 2 summarizes full results for each group. All statistically significant results remained unchanged when analyses were re-performed after removing potential outliers (Supplementary Table S6).

Subsequent frequentist comparison of iADs with vs. without anxiety disorders showed no statistically significant differences. Full results are provided in Supplementary Table S7 (also see Supplementary Figure S1b-f). Secondary analyses comparing iSUDs with vs. without affective disorders are summarized in Supplementary Table S8. These showed significantly higher pessimism (i.e., indicating less directed exploration; *F*(1,130)=5.667, *p*=0.019) and faster learning rate for wins (*F*(1,130)=6.785, *p*=0.010) in iSUDs without affective disorders. Supplementary Figure S2b-f visualizes these results. Also provided in Supplementary Tables S7-S8 are results of analyses after removing potential outliers, confirming that the pattern of results remained unchanged.

### Comparison of within-subject correlations

**Figure 3.**
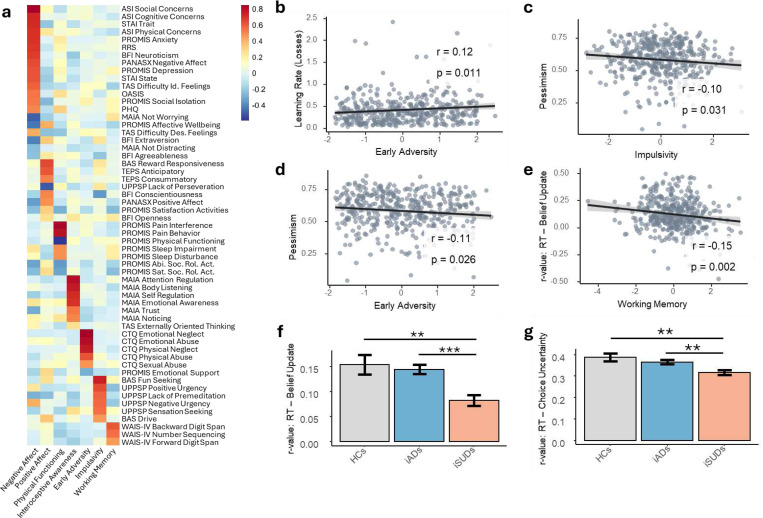
Association between latent factors and computational measures. **a)** Heatmap of factor loadings across measures. The 7 factors were interpreted as reflecting: Negative Affect, Positive Affect, Physical Functioning, Interoceptive Awareness, Early Adversity, Impulsivity, and Working Memory. Detailed descriptions of each measure are provided in Supplementary Materials. **b-e)** Significant relationships between factors and computational parameters. Early Adversity was positively correlated with learning rate for losses. Impulsivity was negatively correlated with pessimism. Working memory was negatively correlated with the Pearson correlation (*r*) values for within-subject associations between reaction times and belief update magnitudes. Note that, while significant at uncorrected thresholds for exploratory purposes, none of these relationships survived correction for 35 comparisons (*p*<.0014). **f-g)** Bar graphs showing the average and standard error of Pearson correlation values reflecting within-subject correlations between reaction times and both belief update magnitudes and choice uncertainty. *p<.05, **p<.01, **p<.001, uncorrected.

One-sample *t*-tests showed that correlations between trial-by-trial reaction times and both belief update magnitude and choice uncertainty were significantly greater than zero across participants (RT-belief update correlations: M=0.12, SD=0.14, *t*(439)=18.231, *p*<0.001; RT-choice uncertainty correlations: M=0.35, SD=0.15, *t*(439)=48.606, *p*<0.001). There were also significant group differences in the magnitude of these correlations (Figure 3f, g). The effect of belief update magnitude (*F*(2,435)=8.654, *p*<0.001, *η*^2^=0.038) indicated that HCs and iADs both had higher correlation values than iSUDs (HCs – iSUDs: c=0.073, *t*=3.232, *p*=0.001; iADs – iSUDs: c=0.056, *t*=3.698, *p*<0.001). The effect of choice uncertainty (*F*(2,435)=6.218, *p*=0.002) similarly indicated significantly higher correlations in HCs and iADs compared to iSUDs (HCs – iSUDs: *c*=0.075, *t*=3.129, *p*=0.002; iADs – iSUDs: *c*=0.044, *t*=2.748, *p*=0.006). Full results are provided in Supplementary Table S9.

### Exploratory analysis of relationships to latent symptom dimensions

Seven factors were identified by the latent factor analysis on dimensional measures. Based on factor loadings (Figure 3a), we label these factors as: Negative Affect, Positive Affect, Physical Functioning, Interoceptive Awareness, Early Adversity, Impulsivity, and Working Memory.

Group comparisons on factor scores showed: 1) higher negative affect and lower positive affect in both clinical groups compared to HCs; 2) lower interoceptive awareness in iADs compared to both HCs and iSUDs; and 3) greater impulsivity in iSUDs than iADs (full results in Supplementary Table S10). Linear models accounting for age and sex revealed significant associations (at uncorrected levels) between these factors and model parameters (Figure 3b-e). Greater pessimism was associated with both lower impulsivity (*F*(1,416)=6.219, *p*=0.013, *b*=−0.015) and less early adversity (*F*(1,416)=3.961, *p*=0.047, *b*=−0.013). Faster learning rates for losses were also associated with greater early adversity (*F*(1,416)=5.819, *p*=0.016, *b*=0.037). However, none of these relationships survived a Bonferroni correction for 35 comparisons (threshold: *p*<.0014). Within-subject correlation values between reaction times and belief update magnitudes were also negatively associated with the working memory factor (*F*(1,413)=10.049, *p*=0.002, *b*=−0.019); that is, those with low working memory had a greater sensitivity to surprising outcomes (Figure 3f,g). Full results are provided in Supplementary Table S11.

When restricting to iADs, greater early adversity was associated with a faster learning rate for losses (*F*(1,221)=4.248, *p*=0.040, *b*=0.049), and less positive affect was associated with greater pessimism (i.e., less exploration drive; *F*(1,221)=4.051, *p*=0.045, *b*=−0.018). When restricting to iSUDs, higher impulsivity was associated with lower pessimism (i.e., greater exploration drive; *F*(1,132)=5.557, *p*=0.020, *b*=−0.026). Restricting to HCs, greater negative affect was associated with higher pessimism (*F*(1,43)=6.546, *p*=0.014, *b*=0.047) and more positive affect was associated with both faster learning rates for wins (*F*(1,43)=5.073, *p*=0.029, *b*=0.082) and greater pessimism (*F*(1,43)=4.901, *p*=0.032, *b*=0.039). Less positive affect was also associated with greater initial decision noise (*F*(1,43)=5.889, *p*=0.020, *b*=−0.070). However, none of these relationships survive correction for multiple comparisons and are reported to motivate replication studies.

## Discussion

This study aimed to investigate whether computational mechanisms associated with learning and decision-making under uncertainty are affected similarly in individuals with affective disorders and substance use disorders compared to healthy controls, and to identify mechanisms that differentiate these clinical groups. Both Bayesian and frequentist analyses indicated slower learning rates for losses in iSUDs compared to iADs, and higher forgetting rates in both iADs and iSUDs compared to HCs. Taken together, these findings suggest distinct cognitive profiles between iADs and iSUDs, with specific impairments in learning and decision-making processes that could inform tailored interventions. Notably, model-free behavioral measures showed no differences between groups, highlighting a crucial role for computational modeling in identifying these patterns Thus, this study underscores the importance of distinguishing between different psychopathological mechanisms to enhance our understanding and treatment of affective and substance use disorders.

In our computational model, forgetting rates lead to a recency bias where decision-making is guided more by recent than remote choice outcomes. This can lead an individual to “jump to conclusions” after one unexpected observation, and it implies the belief that action-outcome relationships change unpredictably over time (i.e., such that patterns in more remote experience should no longer be trusted). Previous work has shown a similar pattern of faster changes in belief in those with high stress/anxiety ^14–18^, often taken to imply that anxious individuals expect the world to be unpredictable. Importantly, however, we also find elevated forgetting rates in those with depression and iSUDs, regardless of their comorbidity with anxiety. Thus, our results could imply that over-reliance on recent experience is a broader, transdiagnostic phenomenon, stemming from a general lack of trust in the ability of previous experience to guide current choices.

This stands in contrast to the difference seen between clinical groups in learning rates for negative outcomes. Unlike typical reinforcement learning approaches, our model distinguishes learning and forgetting. This stems from the additional Bayesian element of our model, which allows an individual to keep track of their confidence in currently expected reward probabilities. While forgetting rates decrease this level of confidence over time, learning rates instead control how strongly a new observation of a given type impacts one’s expected reward probabilities (e.g., which could arise due to factors such as selective attention). Thus, our results here suggest that iADs update their beliefs more quickly in response to negative outcomes than iSUDs, even if confidence in beliefs in general decreases more quickly for both groups relative to HCs. Slower learning from negative outcomes is well-established in iSUDs across many studies for a review, see ^12^, and in this sample specifically ^20^. However, the present findings establish that this form of sensitivity to losses differentiates iSUDs from those with multiple other affective disorders and could therefore reflect a differential vulnerability factor or a consequence of substance use. This appears consistent with recent meta-analytic work in reinforcement learning showing greater punishment learning rates and lower reward learning rates in patients with depression and/or anxiety ^19^. While we do not find faster learning rates from negative outcomes in iADs than HCs in our sample, these prior results would suggest they should not be slower in the manner observed in iSUDs.

Here it is also important to note that, by distinguishing learning and forgetting, it may provide additional granularity when considering previous results using reinforcement learning models. This is because higher learning rates in reinforcement learning cause both faster learning and more forgetting (i.e., they generate the same recency bias as forgetting rates in our model). So prior reinforcement learning studies showing faster learning rates in anxiety ^14–18^, and faster learning from negative outcomes in both depression and anxiety ^19^, could correspond to the forgetting component in our model, while the aforementioned studies showing slower learning from (and generally less sensitivity to) negative outcomes in iSUDs may better map onto the learning rate component of our model. This may be important clinically, as learning vs. forgetting in our model might be modified by different interventions. For example, learning rate might be elevated by increasing attention to a particular outcome that is currently being ignored or explained away, while forgetting may be reduced by helping individuals to see the world as more stable and to avoid jumping to conclusions based on single unexpected outcomes.

Another notable result of our study came from testing whether trial-by-trial metrics of confidence in our model could predict reaction times (i.e., as the model was not fit to this data). In support of the validity of the model, higher confidence did correspond to significant correlations across participants (i.e., more confidence indicating faster decisions); yet, this relationship was itself weaker in iSUDs compared to the other groups. This might imply less sensitivity to uncertainty in iSUDs, and potentially promote non-reflective or impulsive choice. This is consistent with some other studies showing direct or indirect metrics of reduced uncertainty sensitivity in this population (e.g., ^30,31^). However, it should be noted that this did not correlate with measures of impulsivity in our data. Along similar lines, exploratory analyses of relationships between dimensional measures and model parameters showed expected group differences in general negative/positive affect, impulsivity, and interoception consistent with previous literature e.g., see ^58,59–65^, and suggested potential associations between specific parameters and both early adversity and impulsivity. However, these relationships were not hypothesized and did not survive correction for multiple comparisons. They should therefore be replicated in future work before further interpretations or implications are considered.

Other findings were less consistent across analytic approaches or should otherwise be afforded less confidence. For example, iSUDs showed more initial decision noise than iADs in frequentist but not Bayesian analyses. Similarly, Bayesian but not frequentist analyses suggested greater learning rates and initial decision noise in anxious vs. non-anxious depression. While noisier choices in iSUDs and faster learning rates in anxiety disorders are both generally consistent with the prior literature reviewed above, we do not offer strong interpretations here and highlight that these results were generally weaker and should be viewed with caution.

Certain study limitations should be considered. First, iADs had a range of anxiety disorders, and so we cannot differentiate whether one or another anxiety disorder contributed most to our results. That said, all major results did not differ in iADs with vs. without any anxiety disorder. Similarly, iSUDs included those who used a range of substances. However, previous analysis of this and other data suggest common findings across specific disorders ^21^. Another limitation is an imbalance in sample size and sex ratio between groups, with more female iADs in particular. Sex was accounted for in all models, but this does not rule out the possibility that results would differ if males with affective disorders were better represented.

In summary, we find that elevated forgetting rates appear to be a transdiagnostic factor in affective and substance use disorders, while slower learning from negative outcomes differentiates those with vs. without substance use disorders. This takes an important step toward understanding common vs. differential diagnostic mechanisms and highlights granular and distinct mechanisms that could act as treatment targets. These findings are exploratory and will need to be replicated. If confirmed in future work, studies should examine the degree to which they can be modified and how this might influence symptoms in different disorder populations.

## Supplementary Material

Supplement 1

## Figures and Tables

**Table 1:** Demographic and Clinical Sample Characteristics

Variable	HCs (n=54)	iADs (n=242)	iSUDs (n=147)	Test Statistics	*η* ^2^	Post-hoc Contrasts
Age	32.27 (11.35)	35.72 (11.36)	34.05 (9.17)	*F*(2,440)=2.758, *p*=0.065	0.012	n.s.
Sex (Male)	24 (44.44%)	67 (27.69%)	72 (48.98%)	*χ*^2^ =19.377, ***p*<0.001**	--	--
DAST	0.11 (0.37)	0.67 (1.43)	7.54 (2.22)	*F*(2,440)=864.731, ***p*<0.001**	0.797	HCs – iADs, *c*=−0.558, *t*=−2.230, ***p*=0.026** HCs – iSUDs, *c*=−7.426, *t*=−28.057, ***p*<0.001** iADs – iSUDs, *c*=−6.868, *t*=−39.484, ***p*<0.001**
PHQ	0.8 (1.28)	12.93 (4.91)	6.57 (5.68)	*F*(2,440)=170.040, ***p*<0.001**	0.436	HCs – iADs, *c*=−12.138, *t*=−16.430, ***p*<0.001** HCs - iSUDs, *c*=−5.775, *t*=−7.394, ***p*<0.001** iADs – iSUDs, *c*=6.362, *t*=12.395, ***p*<0.001**
OASIS	1.35 (1.94)	9.76 (3.44)	5.84 (4.63)	*F*(2,440)=131.011, ***p*<0.001**	0.373	HCs – iADs, *c*=−8.404, *t*=−14.912, ***p*<0.001** HCs – iSUDs, *c*=−4.492, *t*=−7.538, ***p*<0.001** AD – iSUDs, *c*=3.913, *t*=9.992, ***p*<0.001**
WRAT Reading	63.4 (4.97)	62.52 (5.25)	58.54 (5.9)	*F*(2,409)=27.009, ***p*<0.001**	0.117	HCs – iSUDs, *c*=4.860, *t*=5.481, ***p*<0.001** iADs – iSUDs, *c*=3.978, *t*=6.729, ***p*<0.001**
Psychiatric Medication	8 (14.81%)	170 (70.25%)	67 (45.58%)	*χ*^2^=63.302, ***p*<0.001**	--	--

* Results of analyses of variance (ANOVAs) or chi-squared analyses testing for potential differences between groups (HCs, iADs, iSUDs) in each measure. Pair-wise post-hoc contrasts between estimated marginal means (EMMs) for each group were calculated to interpret significant results. The iADs group consisted of 74 individuals with depression alone and 168 individuals with co-morbid depression and anxiety. DAST: Drug Abuse Screening Test, PHQ: Patient Health Questionnaire, OASIS: Overall Anxiety Severity and Impairment Scale.

**Table 2:** Model parameters estimates for each group (Mean (SD))

Variable	HCs	iADs	iSUDs	Test	*η* ^2^	Post-hoc
Initial Decision Noise	0.4 (0.18)	0.38 (0.18)	0.45 (0.27)	*F*(2,438)=4.134, ***p*=0.017**	0.019	iADs – iSUDs, *c*=−0.065, *t*=−2.808, ***p*=0.005**
Pessimism	0.57 (0.12)	0.59 (0.15)	0.57 (0.14)	*F*(2,438)=0.331, *p*=0.719	0.002	n.s.
Learning Rate (Wins)	0.57 (0.25)	0.59 (0.28)	0.6 (0.26)	*F*(2,438)=0.226, *p*=0.797	0.001	n.s.
Learning Rate (Losses)	0.42 (0.34)	0.45 (0.34)	0.36 (0.24)	*F*(2,438)=3.660, ***p*=0.027**	0.016	iADs – iSUDs, *c*=0.090, *t*=2.706, ***p*=0.007**
Forgetting Rate	0.28 (0.17)	0.38 (0.17)	0.35 (0.14)	*F*(2,438)=5.013, ***p*=0.007**	0.022	HCs – iADs, *c*=−0.072, *t*=−3.163, ***p*=0.002** HCs – iSUDs, *c*=−0.060, *t*=−2.510, ***p*=0.012**
