# Supplementary material for: Computational Mechanisms of Learning and Forgetting Differentiate Affective and Substance Use Disorders
Source: Res Sq. 2024 Oct 31:rs.3.rs-4682224. Preprint. [Version 1] doi: 10.21203/rs.3.rs-4682224/v1 (PMC11581052; doi:10.21203/rs.3.rs-4682224/v1)
Supplement: Supplement 1 [file NIHPPRS4682224V1-supplement-1.pdf]

Supplementary Files

This is a list of supplementary files associated with this preprint. Click to download.

- TABMolecularPsychiatrySupplement2.docx
